# Neutralizing Antibodies Targeting HIV-1 gp41

**DOI:** 10.3390/v12111210

**Published:** 2020-10-23

**Authors:** Christophe Caillat, Delphine Guilligay, Guidenn Sulbaran, Winfried Weissenhorn

**Affiliations:** Institut de Biologie Structurale (IBS), University Grenoble Alpes, Commissariat à L’énergie Atomique et Aux Énergies Alternatives (CEA), Centre National de la Recherche Scientifique (CNRS), 38000 Grenoble, France; caillatchristophe@gmail.com (C.C.); Delphine.guilligay@ibs.fr (D.G.); Guidenn.Sulbaran-Machado@ibs.fr (G.S.)

**Keywords:** HIV-1, Env, gp41, MPER, 4E10, 10E8, DH511, VRC42, LN01, PGZL1, HK20, PGT151, VRC34

## Abstract

HIV-1 vaccine research has obtained an enormous boost since the discovery of many broadly neutralizing antibodies (bnAbs) targeting all accessible sites on the HIV-1 envelope glycoprotein (Env). This in turn facilitated high-resolution structures of the Env glycoprotein in complex with bnAbs. Here we focus on gp41, its highly conserved heptad repeat region 1 (HR1), the fusion peptide (FP) and the membrane-proximal external region (MPER). Notably, the broadest neutralizing antibodies target MPER. Both gp41 HR1 and MPER are only fully accessible once receptor-induced conformational changes have taken place, although some studies suggest access to MPER in the close to native Env conformation. We summarize the data on the structure and function of neutralizing antibodies targeting gp41 HR1, FP and MPER and we review their access to Env and their complex formation with gp41 HR1, MPER peptides and FP within native Env. We further discuss MPER bnAb binding to lipids and the role of somatic mutations in recognizing a bipartite epitope composed of the conserved MPER sequence and membrane components. The problematic of gp41 HR1 access and MPER bnAb auto- and polyreactivity is developed in the light of inducing such antibodies by vaccination.

## 1. Introduction

The HIV-1 envelope glycoprotein (Env) is essential for virus entry into target cells to establish infection. Env forms a trimer of heterodimers composed of the receptor-binding subunit gp120 and the fusion protein gp41. The latter anchors the Env trimer to the membrane and catalyzes membrane fusion. The Env trimer is metastable and gp120 binding to the cellular receptor CD4 induces first conformational changes in Env that subsequently allow interaction with chemokine receptors CXCR4 or CCR5 [1], which leads to the activation of gp41-mediated membrane fusion [1,2,3,4]. Ample structural data suggest that the current structures of the Env ectodomain based on the SOSIP design [5,6] represent the native Env prefusion conformation [7,8,9]. The conformation observed in Env SOSIP structures is in agreement with medium resolution structures of membrane-bound Env [10] and membrane-anchored Env solubilized in detergent or nanodiscs [11,12,13]. In addition, an alternative conformation that precedes the SOSIP conformation has been proposed to explain some discrepancies on antibody binding to membrane-anchored versus soluble Env [14,15].

Although no vaccine has yet been developed, that is capable of inducing a broadly neutralizing antibody (bnAb) response much progress has been made to understand the complex antibody response during infection. Notably, the development of new technologies that allow the identification and isolation of bnAbs from donors whose serum has been identified to have potent and broadly neutralizing activity have revolutionized the field [16]. This led to the discovery of a plethora of antibodies targeting many exposed regions of the prefusion Env trimer and in turn accelerated the structural characterization and optimization of Env-based immunogens [17,18]. Key structures of bnAbs target V1/V2 [19,20,21], glycan-V3 [22,23,24], the CD4-binding site [25,26,27,28,29], the fusion peptide [11,30], the gp120/gp41 interface [31,32], the silent face of gp120 [33], the N-terminal region of the gp41 membrane-proximal external region (MPER) [34] as well as C-terminal MPER [35,36,37,38,39,40,41]. Structural biology together with longitudinal studies on B cell linage development has played a central role in understanding the role of somatic hypermutation in the generation of broadly neutralizing antibodies. Despite the advances, vaccine development poses still enormous challenges due to the antigenic diversity, the high Env glycosylation content, the length variability of Env variable loops, long HCDR 3 regions of bnAbs, which occur with only low frequency in naïve B cells, high levels of somatic mutations, bnAb glycan recognition or accommodation and polyreactivity [42,43,44]. Here we review the structural principles of bnAbs targeting gp41, with a specific focus on MPER-specific antibodies, their bipartite epitope composed of the linear MPER epitope and specific and non-specific membrane interaction. Understanding these principles have important implications for MPER-based vaccine development.

## 2. Antibodies Targeting gp41

**Figure 1:**
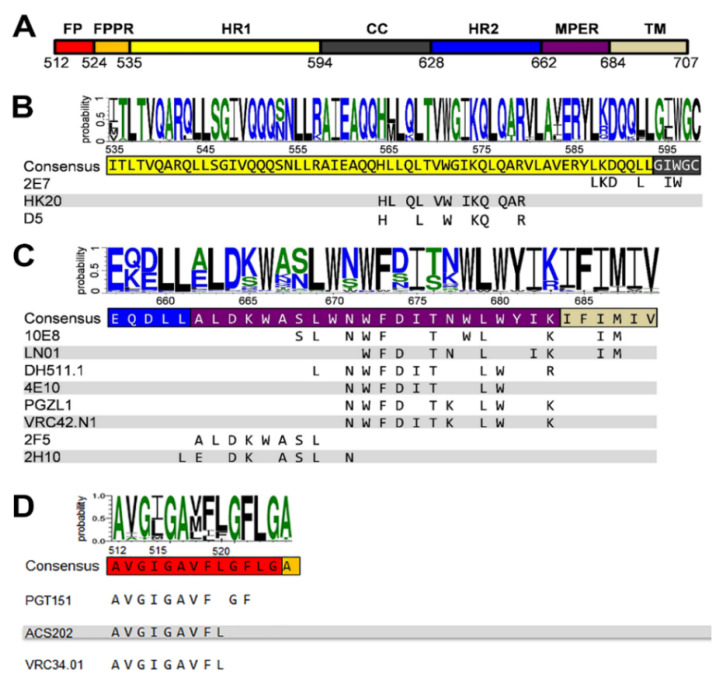
Gp41 sequence conservation and antibody epitopes. Comparison of 5447 sequences of HIV-1; M group (A–K plus recombinants) gp41 sequences are from the sequence database website http://www.hiv.lanl.gov/. (**A**) Organization of gp41 with the different domains: FP, fusion peptide; FPPR, fusion peptide proximal region; HR1, heptad repeat 1; CC, cysteine-linked loop; HR2, heptad repeat 2; MPER, membrane-proximal external region; TM, transmembrane region; Cyt, cytoplasmic domain. Logo showing the amino acid sequence conservation of HR1 (**B**), MPER (**C**) and FP (**D**) [58]. Below the consensus sequence, the epitope of each antibody is shown.

GP41 is a target for vaccine development because of the high sequence conservation of FP, MPER and the heptad repeat region 1 (HR1) (Figure 1A). These gp41 regions are highly conserved (Figure 1B,C) as they play critical roles in membrane fusion including the conformational changes that transform the native prefusion gp41 structure to the gp41 post-fusion conformation [1,3,45]. Notably, MPER contains a tryptophan-rich region that has been implicated in membrane fusion and virus infectivity [46,47]. However, most antibodies induced against gp41 during natural infection are non-neutralizing and target immunodominant regions within the C-C loop [48]. In addition, early anti-gp41 responses are polyreactive revealing cross-reactivity with commensal bacteria of the gut, with cellular proteins and with lipids [49,50,51,52,53]. Thus, the detection of antibodies targeting the linear MPER epitope varies substantially within the different patient cohorts analyzed and neutralizing MPER antibodies are less prevalent than other broadly neutralizing antibodies targeting the native Env trimer [36,54,55,56,57].

## 3. Structure of MPER

Several different MPER conformations have been described based on MPER peptide studies. NMR analyses revealed a kinked MPER structure [59], a continuous helical conformation of MPER fused to the N-terminal part of the transmembrane region (TM) [60] and the complete TM [61]. The physiological role of the continuous helical MPER-TM conformation was confirmed by the structure of the bnAb LN01 in complex with MPER-TM [40]. Furthermore, trimeric models of MPER, TM and MPER-TM have been proposed [62,63,64] and MPER was shown to form a continuous helix with HR2 in the gp41 post-fusion conformation [65,66]. Together, the structural studies indicate the conformational flexibility of MPER with potential hinges within MPER and between MPER and TM. No high-resolution structure of MPER within native Env is yet available, but a recent medium resolution structure suggests that MPER spans approximately 12 Å between the C-terminal gp41 HR2 residue D664 and the membrane boundary at R/K683, thereby forming a “twisting tripod” configuration [10]. As this region contains 19 MPER residues, it remains yet unclear which conformation they might adopt to span this short distance.

## 4. Neutralizing Antibodies Targeting gp41 MPER

**Figure 2:**
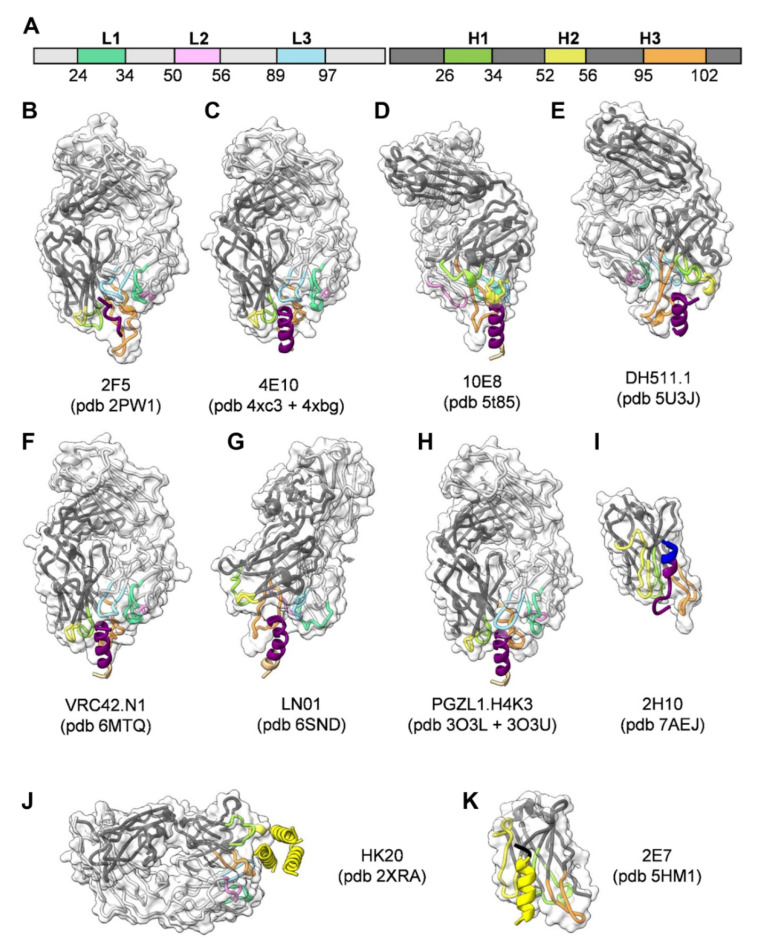
Structures of gp41-specific antibodies in complex with their epitope. The Fabs are represented in ribbon colored according to the light and heavy chain scheme, highlighting the position of the different CDRs (**A**). The surface of the Fabs is represented by a semi-transparent white surface. The gp41 epitope is represented as cartoon colored according to the gp41 scheme shown in Figure 1A. The PBD coordinate files used for generating the figure for each antibody are indicated. (**B**) 2F5; (**C**) 4E10; (**D**) 10E8; (**E**) DH511.1; (**F**) VRC42.N1; (**G**) LN01; (**H**) PGZL1.H4K3; (**I**) llama nanobody 2H10; (**J**) HK20 targeting HR1; (**K**) llama nanobody 2E7 targeting HR1-CC. Images shown in Figure 2, Figure 3 and Figure 4 were rendered by ChimeraX [86].

**Figure 3:**
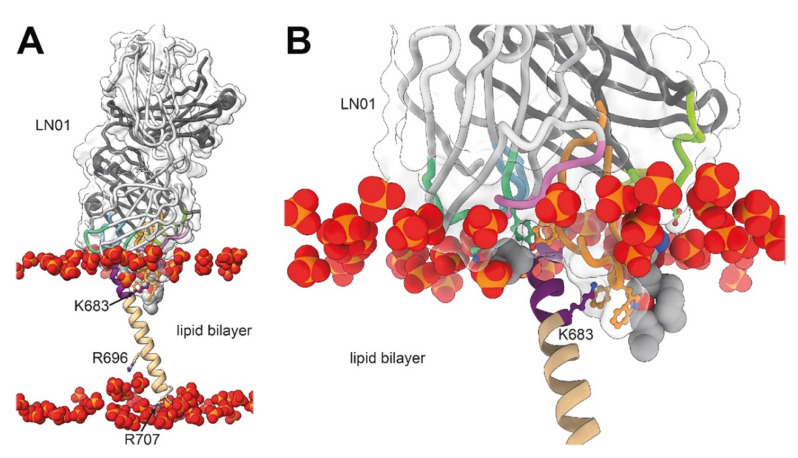
The orientation of the MPER-TM epitope bound to LN01 in the lipid bilayer. (**A**,**B**) Representation of the LN01-MPER-TM complex placed into a bilayer with a lipid composition resembling the viral envelope obtained by molecular dynamics (MD) simulations. The LN01-MPER-TM complex and lipid analogues are represented as shown in Figure 1A and Figure 2A. Only the phosphate group of the lipids of the bilayer are displayed and represented as spheres. (**A**) The tilted orientation of the TM segment with respect to that of the bilayer allows residue K683 to interact with lipid head groups of one leaflet and R696 to contact head groups of the opposite leaflet; residues R707 and R709 terminating the TM helix interact as well with lipid head groups in the simulation. (**B**) Close up of the interaction between LN01, gp41 and the lipids. The gp41 residues located on the same side of the MPER-TM helix as K683 are more accessible to the interaction with the antibody. This orientation of the TM in the lipid bilayer could explain why only one side of the MPER-TM helical epitope (residues 673–686) is targeted by all known MPER bnAbs. The MD simulation supports the interaction of the Fab surface with the bilayer and underlines together with specific lipid-binding shown in later Figures, the recognition of a bipartite epitope composed of MPER and the membrane by MPER bnAbs.

To date, a number of different broadly neutralizing antibodies or antibody lineages thereof have been isolated from patients targeting linear epitopes of MPER (Figure 1C). They are among the broadest neutralizing antibodies in cell-free infection models, but they are less efficient in blocking cell-to-cell transfer [67,68,69]. 2F5 recognizes an extended epitope within N-terminal MPER [34,70] (Figure 2A,B). Additional 2F5-like antibodies m66 and m66.6 have been isolated as well though with a lower extent of somatic mutations and reduced neutralization breadth and potency [71]. Z13E binds to an S-shaped epitope that overlaps with that of 2F5 and 4E10 [37,72]. Abs 4E10 [35,72,73] (Figure 2C), 10E8 [36] (Figure 2D), CAP206-CH12 [74], DH511 [38] (Figure 2E), VRC42 [39] (Figure 2F), LN01 [40] (Figure 2G), and PGZL1 [41] (Figure 2H) recognize linear sequences forming helical epitopes adjacent to the transmembrane region (TM). Recent studies suggest that the MPER epitope extends into the TM since LN01 requires the first helical turn of the TM for interaction [40] and the binding affinity of 10E8 increases in the presence of TM [75], consistent with increased 4E10 binding in the presence of TM [76]. Indeed, the structure of LN01 in complex with MPER fused to the complete TM reveals a continuous helix of the MPER epitope and the TM. Molecular dynamics simulation of the complex within a bilayer resembling the lipid composition of the HIV-1 envelope [77] revealed that MPER-TM is inserted into the membrane with an ~18° tilt, that allows the HCDR3 to dip into the bilayer as well as additional interactions of the Fab surface with the bilayer (Figure 3A,B) [40]. It remains to be determined whether the N-terminal region of the TM is a general feature of the MPER epitope of all anti-MPER antibodies recognizing the helical epitope adjacent to TM. In addition to the isolation of human bnAbs, llama immunization with a gp41 MPER-TM mimetic led to the isolation of the 2H10 nanobody. 2H10 recognizes a 2F5-overlapping epitope (Figure 1C and Figure 2I) and has modest neutralization breadth and potency as a bi-head [78].

MPER is likely sterically occluded within native Env and only accessible upon initial receptor-induced conformational changes [79,80]. Nevertheless, a first encounter complex can be formed with native Env, which however induces conformational changes to facilitate access to the epitope [11,12]. MPER bnAb-Env structures [11,12] suggest further that trimeric MPER [64] needs to “open” up to accommodate bnAb binding. In addition, the different approach angles of MPER bnAbs indicates that MPER is likely in a “monomeric” conformation upon bnAb binding [40]. Furthermore, an induced interaction of Env trimers was likely observed by super-resolution microscopy of MPER bnAb recognition on native virions [81]. This initial interaction with native Env, however, may depend on the neutralization sensitivity of the virus clade. Other studies reveal high-affinity binding to a fusion intermediate gp41 conformation that bridges the viral and cellular membranes [82,83]. Thus, the first encounter with native Env, interaction with early Env conformations and with gp41 intermediates along the fusion pathway suggest that MPER antibodies have a long window of action to inhibit the molecular transitions of gp41 required for entry by membrane fusion. This is in agreement with their relatively long half-life of neutralization. Notably, MPER bnAbs block infection when added up to almost 30 min post-exposure of target cells to HIV-1 [38,84]. Another possible mechanism that contributes to neutralization is their capacity to induce gp120 shedding [80,85]. Moreover, IgG avidity is not required for neutralization, since LN01 Fabs show the same breadth and potency as LN01 IgG1 [40].

## 5. Neutralizing Antibodies Targeting gp41 HR1

Several antibodies targeting the heptad repeat region 1 (HR1) (Figure 1A,B) with modest breadth have been isolated as well. These antibodies target a gp41 pre-hairpin conformation that exposes HR1 and bridges the viral and cellular membranes before refolding into the six-helical bundle conformation required for membrane fusion [87]. The inhibitory action of HR1 targeting Abs is thus comparable to the function of peptide fusion inhibitors [88].

The HR1-specific Fab 3674 was isolated from a human non-immune phage library [89] and further affinity matured (Fab8066) [90]. D5 was selected by vaccination with HR1 mimetics [91] and HK20 was isolated from memory B cells from an infected individual [92]. D5, HK20, and 8066 bind into a conserved hydrophobic pocket of the HR1 triple-stranded coil [93,94,95] (Figure 1A,B and Figure 2J) that is occupied by HR2 in the post-fusion conformation [87,96]. Although D5 and HK20 have modest neutralizing breadth and potency as complete IgGs, the HK20 breadth and potency are greatly increased when used as Fab or scFv, the latter neutralizing 100% out of a 45 pseudovirus panel compared to 15% neutralization by the HK20 IgG [94]. This thus indicates that the size of the complete IgG limits access to this epitope in most clades during the fusion process. Nevertheless, antibodies recognizing the HK20 epitope footprint are present in a significant fraction of HIV-1 infected individuals [94]. Furthermore, it has been shown that the presence of the Fc gamma receptor I (FcγRI) on the target cells substantially increases breadth and potency of mAb D5 and sera from HR1 mimetic immunized guinea pigs neutralizing not only Tier 1 but also Tier-2 viruses from multiple clades in a FcγRI-dependent manner. Whether this is also the case for HK20 needs to be tested. Notably, FcγRI is expressed on macrophages and dendritic cells, which are present at mucosal surfaces and are implicated in the early establishment of HIV-1 infection following sexual transmission, which may reestablish HR1 antibodies as an interesting prevention strategy [97]. An additive effect of FcγRI on neutralization has been also reported for MPER antibodies 2F5 and 4E10 [98,99] in line with the importance of the Fc function of 2F5 for dose-dependent protection of rhesus macaques upon vaginal challenge with SHIV-BaL [100].

Llama immunization with an “open” conformation of HIV-1 Env gp140 CN54 and UG37 led to the isolation of the HR1-targeting nanobody 2E7 [101] that recognizes a C-terminal HR1 epitope extending into the C-C loop of gp41 [102]. In contrast to D5 and HK20, which require coiled-coil interaction for HR1 interaction, 2E7 binds to a linear sequence forming a helical epitope (Figure 1B and Figure 2K). 2E7 neutralizes 80% out of a 26 pseudovirus panel [101] but revealed highly increased breadth and potency when linked to nanobodies recognizing the CD4 binding site [102]. Thus the small size of nanobodies is not only advantageous to penetrate the Env glycan shield [103] but permits also easier access to the HR1 intermediate conformation present in the gp41 pre-hairpin conformation thereby preventing membrane fusion [104].

## 6. Neutralizing Antibodies Targeting gp41 FP

**Figure 4:**
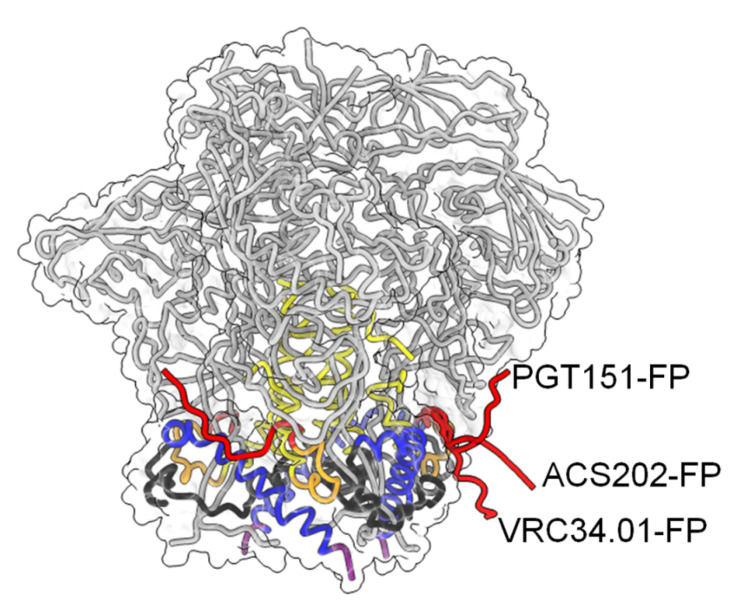
Conformational plasticity of FP. Color-coding of gp41 is as shown in Figure 1A. The Cα trace of gp120 is shown in grey and the molecular envelope of Env is shown transparently. The three different conformations of FP in complex with the indicated bnAbs are shown in red.

Recent work has identified the fusion peptide as a promising target for vaccine development. A number of broadly neutralizing antibodies have been identified that target the conserved FP sequence (Figure 1A,D) at the interface of gp41 and gp120 in the native Env trimer conformation. These include human bnAb PGT151 [105], bnAb VRC34 targeting the N-terminus of FP [30,106] and bnAb ACS202 (Figure 4) [107,108]. In addition to FP, these bnAbs contact other components of the native trimer as well as complex glycans at the gp120-gp41 interface. Notably, immunization schemes employing fusion peptide-coupled carriers combined with Env trimers induced an impressive neutralization of 31% of a cross-clade panel of 208 HIV-1 strains. Isolation of two vaccine-induced murine bnAbs, v1602 and vFP20.01, confirmed the recognition of FP [109] and escape mutants of VRC34 and vaccine-induced bnAbs were mapped to mutations in FP and distal interacting sites of the Env trimer [110]. Together, the structural analyses of Env trimers in complex with FP interacting bnAbs and gp120-gp41 interface bnAbs highlights the conformational plasticity of FP within proteolytically processed native Env trimers [109]. However, FP is not exposed in all native Env trimers and was found to be sequestered in the hydrophobic core of an HIV-1 transmitted founder Env trimer [111]. Nevertheless, immunogens targeting FP have yet produced the broadest cross-clade neutralization by immunization.

## 7. Membrane Interaction and Polyreactivity

**Figure 5:**
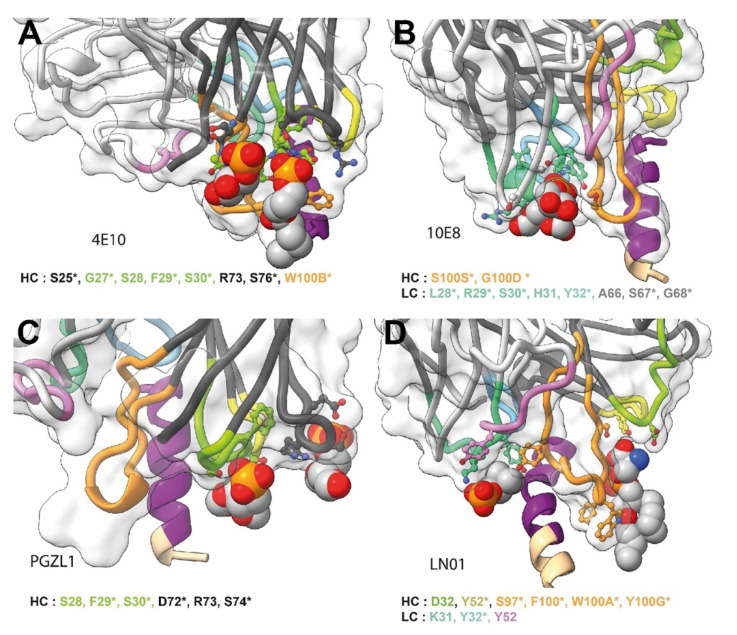
Close-ups of lipid or lipid fragment binding of MPER bnAbs. (**A**) 4E10; (**B**) 10E8; (**C**) LN01 and (**D**) PGZL1. The gp41 epitope and Fabs are represented as in Figure 2. The lipid or lipid fragment visible in the structures are shown as spheres. The residues coordinating the binding of the lipids are shown as stick and balls, the identity and number of these residues are indicated below each structure, an asterisk indicates which residues are present in the UCA.

The hallmark of neutralizing MPER bnAbs is a long HCDR3 region exposing hydrophobic residues at its tip that can insert into the bilayer upon gp41 interaction, which is essential for neutralization [112,113,114,115,116]. In addition, non-specific lipid and membrane interaction in vitro has been observed for 4E10, 2F5 and VRC42.01 [39,52,117]. In contrast, 10E8, DH511 lineage bnAbs and LN01 do not show significant membrane interaction in vitro or with cellular membranes tested by indirect immunofluorescence binding to HEp-2 cells [36,38,40] although some membrane binding using different assays has been reported as well [118]. However, structures of 4E10, 10E8, LN01 and PGZL1 provide evidence for specific lipid binding. 4E10 can bind to lipid components such as glycerol-1-phosphate, glycerol-3-phosphate, phosphatidic acid and phosphatidylglycerol (Figure 5A) [119], 10E8 interacts with glycerol, phosphatidylglycerol and phosphatidic acid (Figure 5B) [120] and LN01 with phosphatidylserine and the phosphocholine group of Fos-choline-12 mimicking a phospholipid interaction (Figure 5C) [40]. Furthermore, PGZL1 and its variant H4K3 coordinate two phosphatidic acid molecules [41] (Figure 5D). To further increase membrane interaction with negatively charged phospholipids basic patches are present in the 10E8 light chain that are positioned at the putative Fab-membrane interface, which are either germline encoded or generated by somatic mutations. Mutations of these basic residues did not affect epitope binding but influenced neutralization. Importantly, mutation of the lipid-binding site abrogated neutralization [120]. Similarly, LN01 variants that lack the lipid-binding site deploy poor neutralization [40]. Like 10E8, LN01 has several basic residues at the Fab-membrane interface that can contact charged lipid head groups [40]. Thus, increasing membrane interaction by engineering additional hydrophobic and basic residues at the Fab-membrane interface augments the potency of 10E8 neutralization [118,121,122,123].

Besides membrane interaction, MPER bnAbs 2F5, 4E10, 10E8 and DH511 lineage bnAbs have been shown to cross-react with cellular proteins [38,124,125]. It was thus suggested that polyreactivity of MPER bnAbs may be the limiting factor to induce such antibodies by vaccination because auto-reactive MPER and membrane-specific B-cells may be eliminated from the B cell repertoire during clonal selection [126,127]. This was strengthened by studying B-cell development in 2F5 and 4E10 knock-in (KI) mice, which showed defects in the transition of pre-B to immature B cells [128,129]. Immunization of 2F5 KI mice with MPER peptide coated liposomes rescued, however, anergic B-cells for neutralizing antibody production, in agreement with some autoreactive B-cells being able to escape clonal selection [130]. In addition, 2F5 germ line KI mice, when immunized with different Env immunogens, induced the deletion of 2F5 precursors, although anergic B cells specific for MPER could be still activated with Env immunogens [131]. This indicates that mechanisms controlling immunological tolerance may limit the generation of 2F5 and 4E10-like bnAbs.

## 8. V-Gene Usage and Somatic Mutations

MPER-specific bnAbs isolated from different patients use members of 6 different VH-genes and 3 different Vκ or Vλ genes. Notably, none of the VH gene usages is rare (Table 1). Most bnAbs targeting Env undergo a large set of somatic mutations to reach breadth and potency. To follow the track of somatic mutations from the unmutated common ancestor (UCA) to the mature bnAb requires samples from different time points of infection, which are often not available complicating the identification of early lineage members. Identification of the UCA and early lineage members would, however, help to design immunogens that can specifically target naïve B-cells expressing UCA receptors.

In case of 10E8, next-generation sequencing (NGS) was used to identify potential early lineage members [132]. Combination with structure-function studies permitted to reconstruct a germline version and early Ab intermediates in the maturation process. This indicated that 10E8 develops from a UCA with no significant MPER binding and substantial differences in HCDR 2 and 3 compared to the mature 10E8. Indeed, structural comparison of unliganded 10E8 with its proposed UCA version and 10E8 with a mature light chain paired with the germline VH revealed significant structural changes of the Cα positions of HCDR 2 and 3 residues. This thus suggests that Ab maturation affects the structure of the HCDRs. Early intermediates showed some weak binding to MPER and modest neutralization, whereas extensive hypermutation is required for broad and potent neutralization [133].

DH511 UCA did not bind to MPER peptides and only the late inferred maturational intermediate, DH511-I6 VH has a detectable affinity for the MPER peptide. Accumulation of neutralization breadth correlates with increased somatic mutations and affinity. Furthermore, in Luminex assay and ELISA, the DH511 UCA reacted with U1 small nuclear RNP (U1-snRNP), and in protein microarray assay, the DH511 UCA was both polyreactive and autoreactive with a number of proteins [38].

A longitudinal study on VRC42 using samples from days 85 to 646 post-infection allowed an accurate reconstruction of the maturation process. This suggests that the Ab becomes broad with only a few somatic mutations starting from day 154. Although the VRC42 UCA (inferred from an early transcript with only 5 nucleotide changes) did not bind to MPER peptides nor does it neutralize, the inferred later intermediate VRC42.I3 neutralized 51% of heterologous viruses with only 13 amino acids different from the inferred VRC42 UCA [39]. Furthermore, VRC42 UCA bound to MPER presented as a multimer on KLH. Although VRC42 showed some degree of polyreactivity and cardiolipin binding, the VRC42 UCA did not interact with HEp-2 cells nor with cardiolipin [39].

No longitudinal information is available on LN01. The inferred LN01 UCA does not interact with MPER peptides. Mixed hc and lc variants having only mature CDRs of LN01 showed that neutralization correlated with binding affinity as expected. Notably, only a few somatic mutations within the CDRs are required for MPER interaction and lipid recognition in the final mature bnAb. Phosphatidylserine (PS) binding is acquired and stabilized by 2 somatic mutations in HCDR1 and while 3 other polar PS interactions from HCDR3 are germline-encoded. Furthermore, LN01 exhibits a low degree of autoreactivity comparable to the one reported for 10E8 [40].

PGZL1 shares germline V/D-region genes with 4E10, but has a shorter CDRH3 and low polyreactivity comparable to 10E8. Notably, a germline revertant of PGZL1 with mature CDR3s still neutralizes 12% of a 130-isolate panel. As an exception, the complete germline reversion of PGZL1 still binds MPER [41] providing a starting point to specifically activate naïve B cells by immunization.

In summary, quite a remarkable number of germline residues are implicated in the binding to the MPER epitope. This implies that only a limited number of somatic mutations are theoretically required to achieve a large breadth and potency. However, long-range structural effects are likely also important for nAb breadth and potency [135]. Of further note, specific lipid binding is not completely germline-encoded and requires somatic mutations for optimal coordination [40,41].

## 9. Gp41 MPER-Based Vaccine Approaches

Although a number of different approaches with different prime-boost strategies have been employed to generate Abs that target MPER (Table 2), none of them showed significant success beyond the induction of some modest neutralization of tier 1 and 2 pseudoviruses. This may be due to multiple reasons. First, polyreactivity as described above may be a major hindrance to generate MPER bnAbs. Thus a hypothesis was put forward suggesting that the clonal lineage UCA of bnAb DH511 may have been initiated by self-antigens while gp41 MPER antigens engaged later to further mature the initial cross-reactive Ab into an anti-MPER bnAb [49,50,51]. Secondly, the immunization strategies followed so far did not take into account an efficient targeting of the naïve B cell receptors. Thirdly, the aspect of the membrane and specific lipids as part of the bipartite epitope has not yet been fully explored, although some approaches presented MPER in a lipid environment. Fourth, the orientation of MPER within a membrane environment needs to be taken into account. This may be a particular problem as the MPER sequence has a high tendency to fold back and interact itself with the membrane thereby occluding its access. Fifth, the lipid composition including cholesterol seems to be important as it may constrain the antigenic conformation of the MPER epitope [136].

## 10. Conclusions

Much progress in understanding the molecular details of bnAbs targeting HR1, FP and MPER in conjunction with components of the bilayer have been made. Notably, FP-specific bnAb generation by immunization is currently the most promising approach. However, regarding MPER as an immunogen, a number of questions are still open. First, which is the gp41 conformation that is finally locked by MPER bnAbs: an early fusion-activated conformation, close to that of native Env, a fusion-intermediate extended conformation or others or all together? Second, can broad and potent MPER-specific bnAbs be induced by vaccination by overcoming high somatic hypermutation and autoreactivity? The findings that bnAbs VRC42, LN01 and especially PGZL1 may require relatively few somatic mutations to acquire high neutralizing activity provides some clues for feasibility. Furthermore, direct lipid binding is not germline-encoded but partly acquired by somatic mutations, suggesting that MPER recognizing germline B-cell receptors may not be clonally deleted because of membrane autoreactivity. Finally, among the first MPER bnAbs, the UCA of PGLZ1 recognizes MPER providing a blueprint to develop vaccination protocols that employ a series of potent immunogens that can first activate naïve B cells and subsequently mature antibodies into high-affinity bnAbs recognizing the bipartite MPER peptide and lipid bilayer epitopes.

## Figures and Tables

**Table 1:** VH and VL gene usage by MPER bnAbs.

bNAb	*VH-Gene*	*VL-Gene*	*D-Segment*	*VH Gene Usage* (%) *
4E10	IGVH1-69	IGVκ3-20*01	D3-10	~2
PGZL1	IGVH1-69	IGVκ3-20	D3-10*01	~2
VRC42	IGVH1-69	IGVκ3-20	D3-10	~2
CAP206-CH12	IGVH1-69	IGVκ3-20*01	n.i.	~2
2F5	IGVH2-05	IGVκ1-13*02	D3-03*01	~1.9
10E8	IGVH3-15	IGVλ3-19	D3-03*01	~2.8
DH511	IGVH3-15	IGVκ1-39	n.i.	~2.8
DH517	IGVH4-34	IGVλ3-19	n.i.	~2.8
M46	IGVH4-34*01	IGVκ1-9*01	D5-12*01	~2.8
LN01	IGVH4-39	IGVκ1-39	D3-3*01	~2.3
Z13e1	IGVH4-59*03	IGVκ3-11*01	n.i.	~8
M44	IGVH4-61*01	IGVκ3-20*01	D3-10*02	~8

* VH gene usage in humans [134]; n.i.: not identified.

**Table 2:** MPER immunogens that elicit modest neutralizing antibody responses in animal models.

Immunogen	Reference
MPER-TM proteoliposomes	[136]
His-tagged MPER bound to liposomes	[137]
Trimeric MPER fused to the diphtheria toxin domainA	[138]
A peptide containing 4 copies of the 10E8 epitope	[139]
Three different 6-helical bundle gp41 constructs containing different bundle destabilizing mutations	[140]
MPER peptide associated with liposomes	[141]
Chimeric human Rhinoviruses expressing MPER	[142]
The MPER and gp41 ectodomain was expressed separately as N-terminal fusions to the E2 protein of *Geobacillus stearothermophilus*; Immunization in conjunction with DNA encoding full-length SF162 gp160	[143]
Hybrid antigens containing the MPER and the FPPR of gp41 of HIV-1 and sequences of the TM protein p15E of PERV	[144]
Tri-repeat of the MPER epitope of gp41 plus defending containing proteoliposomes	[145]
Fusion intermediate conformation of gp41 covalently linked to liposomes	[83]
Gp41 HR2-MPER-TM proteoliposomes	[78]
Bovine papillomavirus VLPs with extended 2F5 or 4E10 epitopes or the MPER domain grafted into the D-E loop of BPV L1	[146]
A HEK293 cell line expressing membrane-anchored gp41	[147]
Gp41-subunit antigens grafted onto liposomes/ virosomes	[148]
Gp140 oligomer prime followed by MPER peptide-liposome boost	[149]
Trimeric MPER fusion proteins	[150]
Gp41 ectodomain fused to an influenza HA2 region	[151]

## References

[1] Chen B. (2019). Molecular Mechanism of HIV-1 Entry. Trends Microbiol..

[2] Schibli D.J., Weissenhorn W. (2006). Class I and class II viral fusion protein structures reveal similar principles in membrane fusion. Mol. Membr. Biol..

[3] Harrison S.C. (2008). Viral membrane fusion. Nat. Struct. Mol. Biol..

[4] Blumenthal R., Durell S., Viard M. (2012). HIV entry and envelope glycoprotein-mediated fusion. J. Biol. Chem..

[5] Sanders R.W., Vesanen M., Schuelke N., Master A., Schiffner L., Kalyanaraman R., Paluch M., Berkhout B., Maddon P.J., Olson W.C. (2002). Stabilization of the soluble, cleaved, trimeric form of the envelope glycoprotein complex of human immunodeficiency virus type 1. J. Virol..

[6] Klasse P.J., Depetris R.S., Pejchal R., Julien J.P., Khayat R., Lee J.H., Marozsan A.J., Cupo A., Cocco N., Korzun J. (2013). Influences on trimerization and aggregation of soluble, cleaved HIV-1 SOSIP envelope glycoprotein. J. Virol..

[7] Lyumkis D., Julien J.P., de Val N., Cupo A., Potter C.S., Klasse P.J., Burton D.R., Sanders R.W., Moore J.P., Carragher B. (2013). Cryo-EM Structure of a Fully Glycosylated Soluble Cleaved HIV-1 Envelope Trimer. Science.

[8] Julien J.P., Cupo A., Sok D., Stanfield R.L., Lyumkis D., Deller M.C., Klasse P.J., Burton D.R., Sanders R.W., Moore J.P. (2013). Crystal Structure of a Soluble Cleaved HIV-1 Envelope Trimer. Science.

[9] Pancera M., Zhou T., Druz A., Georgiev I.S., Soto C., Gorman J., Huang J., Acharya P., Chuang G.Y., Ofek G. (2014). Structure and immune recognition of trimeric pre-fusion HIV-1 Env. Nature.

[10] Li Z., Li W., Lu M., Bess J., Chao C.W., Gorman J., Terry D.S., Zhang B., Zhou T., Blanchard S.C. (2020). Subnanometer structures of HIV-1 envelope trimers on aldrithiol-2-inactivated virus particles. Nat. Struct. Mol. Biol..

[11] Lee J.H., Ozorowski G., Ward A.B. (2016). Cryo-EM structure of a native, fully glycosylated, cleaved HIV-1 envelope trimer. Science.

[12] Rantalainen K., Berndsen Z.T., Antanasijevic A., Schiffner T., Zhang X., Lee W.H., Torres J.L., Zhang L., Irimia A., Copps J. (2020). HIV-1 Envelope and MPER Antibody Structures in Lipid Assemblies. Cell Rep..

[13] Pan J., Peng H., Chen B., Harrison S.C. (2020). Cryo-EM Structure of Full-length HIV-1 Env Bound With the Fab of Antibody PG16. J. Mol. Biol..

[14] Lu M., Ma X., Castillo-Menendez L.R., Gorman J., Alsahafi N., Ermel U., Terry D.S., Chambers M., Peng D., Zhang B. (2019). Associating HIV-1 envelope glycoprotein structures with states on the virus observed by smFRET. Nature.

[15] Wang Q., Finzi A., Sodroski J. (2020). The Conformational States of the HIV-1 Envelope Glycoproteins. Trends Microbiol..

[16] Kwong P.D., Mascola J.R. (2018). HIV-1 Vaccines Based on Antibody Identification, B Cell Ontogeny, and Epitope Structure. Immunity.

[17] Kwong P.D. (2017). What Are the Most Powerful Immunogen Design Vaccine Strategies? A Structural Biologist’s Perspective. Cold Spring Harb. Perspect. Biol..

[18] Ward A.B., Wilson I.A. (2020). Innovations in structure-based antigen design and immune monitoring for next generation vaccines. Curr. Opin. Immunol..

[19] Lee J.H., Andrabi R., Su C.Y., Yasmeen A., Julien J.P., Kong L., Wu N.C., McBride R., Sok D., Pauthner M. (2017). A Broadly Neutralizing Antibody Targets the Dynamic HIV Envelope Trimer Apex via a Long, Rigidified, and Anionic beta-Hairpin Structure. Immunity.

[20] McLellan J.S., Pancera M., Carrico C., Gorman J., Julien J.P., Khayat R., Louder R., Pejchal R., Sastry M., Dai K. (2011). Structure of HIV-1 gp120 V1/V2 domain with broadly neutralizing antibody PG9. Nature.

[21] Cale E.M., Gorman J., Radakovich N.A., Crooks E.T., Osawa K., Tong T., Li J., Nagarajan R., Ozorowski G., Ambrozak D.R. (2017). Virus-like Particles Identify an HIV V1V2 Apex-Binding Neutralizing Antibody that Lacks a Protruding Loop. Immunity.

[22] Pejchal R., Doores K.J., Walker L.M., Khayat R., Huang P.S., Wang S.K., Stanfield R.L., Julien J.P., Ramos A., Crispin M. (2011). A potent and broad neutralizing antibody recognizes and penetrates the HIV glycan shield. Science.

[23] Julien J.P., Sok D., Khayat R., Lee J.H., Doores K.J., Walker L.M., Ramos A., Diwanji D.C., Pejchal R., Cupo A. (2013). Broadly neutralizing antibody PGT121 allosterically modulates CD4 binding via recognition of the HIV-1 gp120 V3 base and multiple surrounding glycans. PLoS Pathog..

[24] Kong L., Lee J.H., Doores K.J., Murin C.D., Julien J.P., McBride R., Liu Y., Marozsan A., Cupo A., Klasse P.J. (2013). Supersite of immune vulnerability on the glycosylated face of HIV-1 envelope glycoprotein gp120. Nat. Struct. Mol. Biol..

[25] Liao H.X., Lynch R., Zhou T., Gao F., Alam S.M., Boyd S.D., Fire A.Z., Roskin K.M., Schramm C.A., Zhang Z. (2013). Co-evolution of a broadly neutralizing HIV-1 antibody and founder virus. Nature.

[26] Zhou T., Xu L., Dey B., Hessell A.J., Van Ryk D., Xiang S.H., Yang X., Zhang M.Y., Zwick M.B., Arthos J. (2007). Structural definition of a conserved neutralization epitope on HIV-1 gp120. Nature.

[27] Zhou T., Lynch R.M., Chen L., Acharya P., Wu X., Doria-Rose N.A., Joyce M.G., Lingwood D., Soto C., Bailer R.T. (2015). Structural Repertoire of HIV-1-Neutralizing Antibodies Targeting the CD4 Supersite in 14 Donors. Cell.

[28] Zhou T., Georgiev I., Wu X., Yang Z.Y., Dai K., Finzi A., Kwon Y.D., Scheid J.F., Shi W., Xu L. (2010). Structural basis for broad and potent neutralization of HIV-1 by antibody VRC01. Science.

[29] Wu X., Zhou T., Zhu J., Zhang B., Georgiev I., Wang C., Chen X., Longo N.S., Louder M., McKee K. (2011). Focused evolution of HIV-1 neutralizing antibodies revealed by structures and deep sequencing. Science.

[30] Kong R., Xu K., Zhou T., Acharya P., Lemmin T., Liu K., Ozorowski G., Soto C., Taft J.D., Bailer R.T. (2016). Fusion peptide of HIV-1 as a site of vulnerability to neutralizing antibody. Science.

[31] Scharf L., Scheid J.F., Lee J.H., West A.P., Chen C., Gao H., Gnanapragasam P.N., Mares R., Seaman M.S., Ward A.B. (2014). Antibody 8ANC195 reveals a site of broad vulnerability on the HIV-1 envelope spike. Cell Rep..

[32] Huang J., Kang B.H., Pancera M., Lee J.H., Tong T., Feng Y., Imamichi H., Georgiev I.S., Chuang G.Y., Druz A. (2014). Broad and potent HIV-1 neutralization by a human antibody that binds the gp41-gp120 interface. Nature.

[33] Zhou T., Zheng A., Baxa U., Chuang G.Y., Georgiev I.S., Kong R., O’Dell S., Shahzad-Ul-Hussan S., Shen C.H., Tsybovsky Y. (2018). A Neutralizing Antibody Recognizing Primarily N-Linked Glycan Targets the Silent Face of the HIV Envelope. Immunity.

[34] Ofek G., Tang M., Sambor A., Katinger H., Mascola J.R., Wyatt R., Kwong P.D. (2004). Structure and mechanistic analysis of the anti-human immunodeficiency virus type 1 antibody 2F5 in complex with its gp41 epitope. J. Virol..

[35] Cardoso R.M., Zwick M.B., Stanfield R.L., Kunert R., Binley J.M., Katinger H., Burton D.R., Wilson I.A. (2005). Broadly neutralizing anti-HIV antibody 4E10 recognizes a helical conformation of a highly conserved fusion-associated motif in gp41. Immunity.

[36] Huang J., Ofek G., Laub L., Louder M.K., Doria-Rose N.A., Longo N.S., Imamichi H., Bailer R.T., Chakrabarti B., Sharma S.K. (2012). Broad and potent neutralization of HIV-1 by a gp41-specific human antibody. Nature.

[37] Pejchal R., Gach J.S., Brunel F.M., Cardoso R.M., Stanfield R.L., Dawson P.E., Burton D.R., Zwick M.B., Wilson I.A. (2009). A Conformational Switch in Human Immunodeficiency Virus gp41 Revealed by the Structures of Overlapping Epitopes Recognized by Neutralizing Antibodies. J. Virol..

[38] Williams L.D., Ofek G., Schatzle S., McDaniel J.R., Lu X., Nicely N.I., Wu L., Lougheed C.S., Bradley T., Louder M.K. (2017). Potent and broad HIV-neutralizing antibodies in memory B cells and plasma. Sci. Immunol..

[39] Krebs S.J., Kwon Y.D., Schramm C.A., Law W.H., Donofrio G., Zhou K.H., Gift S., Dussupt V., Georgiev I.S., Schatzle S. (2019). Longitudinal Analysis Reveals Early Development of Three MPER-Directed Neutralizing Antibody Lineages from an HIV-1-Infected Individual. Immunity.

[40] Pinto D., Fenwick C., Caillat C., Silacci C., Guseva S., Dehez F., Chipot C., Barbieri S., Minola A., Jarrossay D. (2019). Structural Basis for Broad HIV-1 Neutralization by the MPER-Specific Human Broadly Neutralizing Antibody LN01. Cell Host Microbe.

[41] Zhang L., Irimia A., He L., Landais E., Rantalainen K., Leaman D.P., Vollbrecht T., Stano A., Sands D.I., Kim A.S. (2019). An MPER antibody neutralizes HIV-1 using germline features shared among donors. Nat. Commun..

[42] Sok D., Burton D.R. (2018). Recent progress in broadly neutralizing antibodies to HIV. Nat. Immunol..

[43] Haynes B.F., Burton D.R., Mascola J.R. (2019). Multiple roles for HIV broadly neutralizing antibodies. Sci. Transl. Med..

[44] Stephenson K.E., Wagh K., Korber B., Barouch D.H. (2020). Vaccines and Broadly Neutralizing Antibodies for HIV-1 Prevention. Annu. Rev. Immunol..

[45] Weissenhorn W., Hinz A., Gaudin Y. (2007). Virus membrane fusion. FEBS Lett..

[46] Munoz-Barroso I., Salzwedel K., Hunter E., Blumenthal R. (1999). Role of the membrane-proximal domain in the initial stages of human immunodeficiency virus type 1 envelope glycoprotein-mediated membrane fusion. J. Virol..

[47] Salzwedel K., West J.T., Hunter E. (1999). A conserved tryptophan-rich motif in the membrane-proximal region of the human immunodeficiency virus type 1 gp41 ectodomain is important for Env-mediated fusion and virus infectivity. J. Virol..

[48] Tomaras G.D., Yates N.L., Liu P., Qin L., Fouda G.G., Chavez L.L., Decamp A.C., Parks R.J., Ashley V.C., Lucas J.T. (2008). Initial B-cell responses to transmitted human immunodeficiency virus type 1: Virion-binding immunoglobulin M (IgM) and IgG antibodies followed by plasma anti-gp41 antibodies with ineffective control of initial viremia. J. Virol..

[49] Liao H.X., Chen X., Munshaw S., Zhang R., Marshall D.J., Vandergrift N., Whitesides J.F., Lu X., Yu J.S., Hwang K.K. (2011). Initial antibodies binding to HIV-1 gp41 in acutely infected subjects are polyreactive and highly mutated. J. Exp. Med..

[50] Trama A.M., Moody M.A., Alam S.M., Jaeger F.H., Lockwood B., Parks R., Lloyd K.E., Stolarchuk C., Scearce R., Foulger A. (2014). HIV-1 envelope gp41 antibodies can originate from terminal ileum B cells that share cross-reactivity with commensal bacteria. Cell Host Microbe.

[51] Williams W.B., Liao H.X., Moody M.A., Kepler T.B., Alam S.M., Gao F., Wiehe K., Trama A.M., Jones K., Zhang R. (2015). Diversion of HIV-1 vaccine-induced immunity by gp41-microbiota cross-reactive antibodies. Science.

[52] Haynes B.F., Fleming J., St Clair E.W., Katinger H., Stiegler G., Kunert R., Robinson J., Scearce R.M., Plonk K., Staats H.F. (2005). Cardiolipin polyspecific autoreactivity in two broadly neutralizing HIV-1 antibodies. Science.

[53] Haynes B.F., Moody M.A., Verkoczy L., Kelsoe G., Alam S.M. (2005). Antibody polyspecificity and neutralization of HIV-1: A hypothesis. Hum. Antib..

[54] Braibant M., Brunet S., Costagliola D., Rouzioux C., Agut H., Katinger H., Autran B., Barin F. (2006). Antibodies to conserved epitopes of the HIV-1 envelope in sera from long-term non-progressors: Prevalence and association with neutralizing activity. AIDS.

[55] Gray E.S., Madiga M.C., Moore P.L., Mlisana K., Abdool Karim S.S., Binley J.M., Shaw G.M., Mascola J.R., Morris L. (2009). Broad neutralization of human immunodeficiency virus type 1 mediated by plasma antibodies against the gp41 membrane proximal external region. J. Virol..

[56] Landais E., Huang X., Havenar-Daughton C., Murrell B., Price M.A., Wickramasinghe L., Ramos A., Bian C.B., Simek M., Allen S. (2016). Broadly Neutralizing Antibody Responses in a Large Longitudinal Sub-Saharan HIV Primary Infection Cohort. PLoS Pathog..

[57] Gonzalez N., McKee K., Lynch R.M., Georgiev I.S., Jimenez L., Grau E., Yuste E., Kwong P.D., Mascola J.R., Alcami J. (2018). Characterization of broadly neutralizing antibody responses to HIV-1 in a cohort of long term non-progressors. PLoS ONE.

[58] Crooks G.E., Hon G., Chandonia J.M., Brenner S.E. (2004). WebLogo: A sequence logo generator. Genome Res..

[59] Sun Z.Y., Oh K.J., Kim M., Yu J., Brusic V., Song L., Qiao Z., Wang J.H., Wagner G., Reinherz E.L. (2008). HIV-1 broadly neutralizing antibody extracts its epitope from a kinked gp41 ectodomain region on the viral membrane. Immunity.

[60] Apellaniz B., Rujas E., Serrano S., Morante K., Tsumoto K., Caaveiro J.M., Jimenez M.A., Nieva J.L. (2015). The Atomic Structure of the HIV-1 gp41 Transmembrane Domain and Its Connection to the Immunogenic Membrane-proximal External Region. J. Biol. Chem..

[61] Chiliveri S.C., Louis J.M., Ghirlando R., Baber J.L., Bax A. (2018). Tilted, Uninterrupted, Monomeric HIV-1 gp41 Transmembrane Helix from Residual Dipolar Couplings. J. Am. Chem. Soc..

[62] Reardon P.N., Sage H., Dennison S.M., Martin J.W., Donald B.R., Alam S.M., Haynes B.F., Spicer L.D. (2014). Structure of an HIV-1-neutralizing antibody target, the lipid-bound gp41 envelope membrane proximal region trimer. Proc. Natl. Acad. Sci. USA.

[63] Dev J., Park D., Fu Q., Chen J., Ha H.J., Ghantous F., Herrmann T., Chang W., Liu Z., Frey G. (2016). Structural basis for membrane anchoring of HIV-1 envelope spike. Science.

[64] Fu Q., Shaik M.M., Cai Y., Ghantous F., Piai A., Peng H., Rits-Volloch S., Liu Z., Harrison S.C., Seaman M.S. (2018). Structure of the membrane proximal external region of HIV-1 envelope glycoprotein. Proc. Natl. Acad. Sci. USA.

[65] Shi W., Bohon J., Han D.P., Habte H., Qin Y., Cho M.W., Chance M.R. (2010). Structural characterization of HIV gp41 with the membrane-proximal external region. J. Biol. Chem..

[66] Buzon V., Natrajan G., Schibli D., Campelo F., Kozlov M.M., Weissenhorn W. (2010). Crystal structure of HIV-1 gp41 including both fusion peptide and membrane proximal external regions. PLoS Pathog..

[67] Dufloo J., Bruel T., Schwartz O. (2018). HIV-1 cell-to-cell transmission and broadly neutralizing antibodies. Retrovirology.

[68] Schiffner T., Sattentau Q.J., Duncan C.J. (2013). Cell-to-cell spread of HIV-1 and evasion of neutralizing antibodies. Vaccine.

[69] Malbec M., Porrot F., Rua R., Horwitz J., Klein F., Halper-Stromberg A., Scheid J.F., Eden C., Mouquet H., Nussenzweig M.C. (2013). Broadly neutralizing antibodies that inhibit HIV-1 cell to cell transmission. J. Exp. Med..

[70] Muster T., Guinea R., Trkola A., Purtscher M., Klima A., Steindl F., Palese P., Katinger H. (1994). Cross-neutralizing activity against divergent human immunodeficiency virus type 1 isolates induced by the gp41 sequence ELDKWAS. J. Virol..

[71] Ofek G., Zirkle B., Yang Y., Zhu Z., McKee K., Zhang B., Chuang G.Y., Georgiev I.S., O’Dell S., Doria-Rose N. (2014). Structural basis for HIV-1 neutralization by 2F5-like antibodies m66 and m66.6. J. Virol..

[72] Zwick M.B., Labrijn A.F., Wang M., Spenlehauer C., Saphire E.O., Binley J.M., Moore J.P., Stiegler G., Katinger H., Burton D.R. (2001). Broadly neutralizing antibodies targeted to the membrane-proximal external region of human immunodeficiency virus type 1 glycoprotein gp41. J. Virol..

[73] Stiegler G., Kunert R., Purtscher M., Wolbank S., Voglauer R., Steindl F., Katinger H. (2001). A potent cross-clade neutralizing human monoclonal antibody against a novel epitope on gp41 of human immunodeficiency virus type 1. AIDS Res. Hum. Retrovir..

[74] Morris L., Chen X., Alam M., Tomaras G., Zhang R., Marshall D.J., Chen B., Parks R., Foulger A., Jaeger F. (2011). Isolation of a human anti-HIV gp41 membrane proximal region neutralizing antibody by antigen-specific single B cell sorting. PLoS ONE.

[75] Rujas E., Caaveiro J.M., Partida-Hanon A., Gulzar N., Morante K., Apellaniz B., Garcia-Porras M., Bruix M., Tsumoto K., Scott J.K. (2016). Structural basis for broad neutralization of HIV-1 through the molecular recognition of 10E8 helical epitope at the membrane interface. Sci. Rep..

[76] Montero M., Gulzar N., Klaric K.A., Donald J.E., Lepik C., Wu S., Tsai S., Julien J.P., Hessell A.J., Wang S. (2012). Neutralizing epitopes in the membrane-proximal external region of HIV-1 gp41 are influenced by the transmembrane domain and the plasma membrane. J. Virol..

[77] Brugger B., Glass B., Haberkant P., Leibrecht I., Wieland F.T., Krausslich H.G. (2006). The HIV lipidome: A raft with an unusual composition. Proc. Natl. Acad. Sci. USA.

[78] Lutje Hulsik D., Liu Y.Y., Strokappe N.M., Battella S., El Khattabi M., McCoy L.E., Sabin C., Hinz A., Hock M., Macheboeuf P. (2013). A gp41 MPER-specific llama VHH requires a hydrophobic CDR3 for neutralization but not for antigen recognition. PLoS Pathog..

[79] Chakrabarti B.K., Walker L.M., Guenaga J.F., Ghobbeh A., Poignard P., Burton D.R., Wyatt R.T. (2011). Direct antibody access to the HIV-1 membrane-proximal external region positively correlates with neutralization sensitivity. J. Virol..

[80] Ruprecht C.R., Krarup A., Reynell L., Mann A.M., Brandenberg O.F., Berlinger L., Abela I.A., Regoes R.R., Gunthard H.F., Rusert P. (2011). MPER-specific antibodies induce gp120 shedding and irreversibly neutralize HIV-1. J. Exp. Med..

[81] Carravilla P., Chojnacki J., Rujas E., Insausti S., Largo E., Waithe D., Apellaniz B., Sicard T., Julien J.P., Eggeling C. (2019). Molecular recognition of the native HIV-1 MPER revealed by STED microscopy of single virions. Nat. Commun..

[82] Frey G., Peng H., Rits-Volloch S., Morelli M., Cheng Y., Chen B. (2008). A fusion-intermediate state of HIV-1 gp41 targeted by broadly neutralizing antibodies. Proc. Natl. Acad. Sci. USA.

[83] Lai R.P.J., Hock M., Radzimanowski J., Tonks P., Hulsik D.L., Effantin G., Seilly D.J., Dreja H., Kliche A., Wagner R. (2014). A Fusion Intermediate gp41 Immunogen Elicits Neutralizing Antibodies to HIV-1. J. Biol. Chem..

[84] Shen X., Dennison S.M., Liu P., Gao F., Jaeger F., Montefiori D.C., Verkoczy L., Haynes B.F., Alam S.M., Tomaras G.D. (2010). Prolonged exposure of the HIV-1 gp41 membrane proximal region with L669S substitution. Proc. Natl. Acad. Sci. USA.

[85] Sattentau Q.J., Moulard M., Brivet B., Botto F., Guillemot J.C., Mondor I., Poignard P., Ugolini S. (1999). Antibody neutralization of HIV-1 and the potential for vaccine design. Immunol. Lett..

[86] Goddard T.D., Huang C.C., Meng E.C., Pettersen E.F., Couch G.S., Morris J.H., Ferrin T.E. (2018). UCSF ChimeraX: Meeting modern challenges in visualization and analysis. Protein Sci..

[87] Weissenhorn W., Dessen A., Harrison S.C., Skehel J.J., Wiley D.C. (1997). Atomic structure of the ectodomain from HIV-1 gp41. Nature.

[88] Su X., Wang Q., Wen Y., Jiang S., Lu L. (2020). Protein- and Peptide-Based Virus Inactivators: Inactivating Viruses before Their Entry into Cells. Front. Microbiol..

[89] Gustchina E., Louis J.M., Lam S.N., Bewley C.A., Clore G.M. (2007). A monoclonal Fab derived from a human nonimmune phage library reveals a new epitope on gp41 and neutralizes diverse human immunodeficiency virus type 1 strains. J. Virol..

[90] Gustchina E., Louis J.M., Frisch C., Ylera F., Lechner A., Bewley C.A., Clore G.M. (2009). Affinity maturation by targeted diversification of the CDR-H2 loop of a monoclonal Fab derived from a synthetic naive human antibody library and directed against the internal trimeric coiled-coil of gp41 yields a set of Fabs with improved HIV-1 neutralization potency and breadth. Virology.

[91] Miller M.D., Geleziunas R., Bianchi E., Lennard S., Hrin R., Zhang H., Lu M., An Z., Ingallinella P., Finotto M. (2005). A human monoclonal antibody neutralizes diverse HIV-1 isolates by binding a critical gp41 epitope. Proc. Natl. Acad. Sci. USA.

[92] Corti D., Langedijk J.P., Hinz A., Seaman M.S., Vanzetta F., Fernandez-Rodriguez B.M., Silacci C., Pinna D., Jarrossay D., Balla-Jhagjhoorsingh S. (2010). Analysis of memory B cell responses and isolation of novel monoclonal antibodies with neutralizing breadth from HIV-1-infected individuals. PLoS ONE.

[93] Luftig M.A., Mattu M., Di Giovine P., Geleziunas R., Hrin R., Barbato G., Bianchi E., Miller M.D., Pessi A., Carfi A. (2006). Structural basis for HIV-1 neutralization by a gp41 fusion intermediate-directed antibody. Nat. Struct. Mol. Biol..

[94] Sabin C., Corti D., Buzon V., Seaman M.S., Lutje Hulsik D., Hinz A., Vanzetta F., Agatic G., Silacci C., Mainetti L. (2010). Crystal structure and size-dependent neutralization properties of HK20, a human antibody binding to the highly conserved heptad repeat 1 of gp41. PLoS Pathog..

[95] Gustchina E., Li M., Louis J.M., Anderson D.E., Lloyd J., Frisch C., Bewley C.A., Gustchina A., Wlodawer A., Clore G.M. (2010). Structural basis of HIV-1 neutralization by affinity matured Fabs directed against the internal trimeric coiled-coil of gp41. PLoS Pathog..

[96] Chan D.C., Fass D., Berger J.M., Kim P.S. (1997). Core structure of gp41 from the HIV envelope glycoprotein. Cell.

[97] Montefiori D.C., Filsinger Interrante M.V., Bell B.N., Rubio A.A., Joyce J.G., Shiver J.W., LaBranche C.C., Kim P.S. (2020). The high-affinity immunoglobulin receptor FcγRI potentiates HIV-1 neutralization via antibodies against the gp41 N-heptad repeat. bioRxiv.

[98] Perez L.G., Costa M.R., Todd C.A., Haynes B.F., Montefiori D.C. (2009). Utilization of immunoglobulin G Fc receptors by human immunodeficiency virus type 1: A specific role for antibodies against the membrane-proximal external region of gp41. J. Virol..

[99] Perez L.G., Zolla-Pazner S., Montefiori D.C. (2013). Antibody-DEPENDENT, FcgammaRI-mediated neutralization of HIV-1 in TZM-bl cells occurs independently of phagocytosis. J. Virol..

[100] Klein K., Veazey R.S., Warrier R., Hraber P., Doyle-Meyers L.A., Buffa V., Liao H.X., Haynes B.F., Shaw G.M., Shattock R.J. (2013). Neutralizing IgG at the portal of infection mediates protection against vaginal simian/human immunodeficiency virus challenge. J. Virol..

[101] Strokappe N., Szynol A., Aasa-Chapman M., Gorlani A., Forsman Quigley A., Hulsik D.L., Chen L., Weiss R., de Haard H., Verrips T. (2012). Llama antibody fragments recognizing various epitopes of the CD4bs neutralize a broad range of HIV-1 subtypes A, B and C. PLoS ONE.

[102] Strokappe N.M., Hock M., Rutten L., McCoy L.E., Back J.W., Caillat C., Haffke M., Weiss R.A., Weissenhorn W., Verrips T. (2019). Super Potent Bispecific Llama VHH Antibodies Neutralize HIV via a Combination of gp41 and gp120 Epitopes. Antibodies (Basel).

[103] Weiss R.A., Verrips C.T. (2019). Nanobodies that Neutralize HIV. Vaccines (Basel).

[104] Weissenhorn W., Dessen A., Calder L., Harrison S., Skehel J., Wiley D. (1999). Structural basis for membrane fusion by enveloped viruses. Mol. Membr. Biol..

[105] Blattner C., Lee J.H., Sliepen K., Derking R., Falkowska E., de la Pena A.T., Cupo A., Julien J.P., van Gils M., Lee P.S. (2014). Structural delineation of a quaternary, cleavage-dependent epitope at the gp41-gp120 interface on intact HIV-1 Env trimers. Immunity.

[106] Shen C.H., DeKosky B.J., Guo Y., Xu K., Gu Y., Kilam D., Ko S.H., Kong R., Liu K., Louder M.K. (2020). VRC34-Antibody Lineage Development Reveals How a Required Rare Mutation Shapes the Maturation of a Broad HIV-Neutralizing Lineage. Cell Host Microbe.

[107] van Gils M.J., van den Kerkhof T.L., Ozorowski G., Cottrell C.A., Sok D., Pauthner M., Pallesen J., de Val N., Yasmeen A., de Taeye S.W. (2016). An HIV-1 antibody from an elite neutralizer implicates the fusion peptide as a site of vulnerability. Nat. Microbiol..

[108] Yuan M., Cottrell C.A., Ozorowski G., van Gils M.J., Kumar S., Wu N.C., Sarkar A., Torres J.L., de Val N., Copps J. (2019). Conformational Plasticity in the HIV-1 Fusion Peptide Facilitates Recognition by Broadly Neutralizing Antibodies. Cell Host Microbe.

[109] Xu K., Acharya P., Kong R., Cheng C., Chuang G.Y., Liu K., Louder M.K., O’Dell S., Rawi R., Sastry M. (2018). Epitope-based vaccine design yields fusion peptide-directed antibodies that neutralize diverse strains of HIV-1. Nat. Med..

[110] Dingens A.S., Acharya P., Haddox H.K., Rawi R., Xu K., Chuang G.Y., Wei H., Zhang B., Mascola J.R., Carragher B. (2018). Complete functional mapping of infection- and vaccine-elicited antibodies against the fusion peptide of HIV. PLoS Pathog..

[111] Ananthaswamy N., Fang Q., AlSalmi W., Jain S., Chen Z., Klose T., Sun Y., Liu Y., Mahalingam M., Chand S. (2019). A sequestered fusion peptide in the structure of an HIV-1 transmitted founder envelope trimer. Nat. Commun..

[112] Zwick M.B., Komori H.K., Stanfield R.L., Church S., Wang M., Parren P.W., Kunert R., Katinger H., Wilson I.A., Burton D.R. (2004). The long third complementarity-determining region of the heavy chain is important in the activity of the broadly neutralizing anti-human immunodeficiency virus type 1 antibody 2F5. J. Virol..

[113] Ofek G., McKee K., Yang Y., Yang Z.Y., Skinner J., Guenaga F.J., Wyatt R., Zwick M.B., Nabel G.J., Mascola J.R. (2010). Relationship between antibody 2F5 neutralization of HIV-1 and hydrophobicity of its heavy chain third complementarity-determining region. J. Virol..

[114] Alam S.M., Morelli M., Dennison S.M., Liao H.X., Zhang R., Xia S.M., Rits-Volloch S., Sun L., Harrison S.C., Haynes B.F. (2009). Role of HIV membrane in neutralization by two broadly neutralizing antibodies. Proc. Natl. Acad. Sci. USA.

[115] Scherer E.M., Leaman D.P., Zwick M.B., McMichael A.J., Burton D.R. (2010). Aromatic residues at the edge of the antibody combining site facilitate viral glycoprotein recognition through membrane interactions. Proc. Natl. Acad. Sci. USA.

[116] Julien J.P., Huarte N., Maeso R., Taneva S.G., Cunningham A., Nieva J.L., Pai E.F. (2010). Ablation of the complementarity-determining region H3 apex of the anti-HIV-1 broadly neutralizing antibody 2F5 abrogates neutralizing capacity without affecting core epitope binding. J. Virol..

[117] Alam S.M., McAdams M., Boren D., Rak M., Scearce R.M., Gao F., Camacho Z.T., Gewirth D., Kelsoe G., Chen P. (2007). The role of antibody polyspecificity and lipid reactivity in binding of broadly neutralizing anti-HIV-1 envelope human monoclonal antibodies 2F5 and 4E10 to glycoprotein 41 membrane proximal envelope epitopes. J. Immunol..

[118] Chen J., Frey G., Peng H., Rits-Volloch S., Garrity J., Seaman M.S., Chen B. (2014). Mechanism of HIV-1 neutralization by antibodies targeting a membrane-proximal region of gp41. J. Virol..

[119] Irimia A., Sarkar A., Stanfield R.L., Wilson I.A. (2016). Crystallographic Identification of Lipid as an Integral Component of the Epitope of HIV Broadly Neutralizing Antibody 4E10. Immunity.

[120] Irimia A., Serra A.M., Sarkar A., Jacak R., Kalyuzhniy O., Sok D., Saye-Francisco K.L., Schiffner T., Tingle R., Kubitz M. (2017). Lipid interactions and angle of approach to the HIV-1 viral membrane of broadly neutralizing antibody 10E8: Insights for vaccine and therapeutic design. PLoS Pathog..

[121] Kwon Y.D., Chuang G.Y., Zhang B., Bailer R.T., Doria-Rose N.A., Gindin T.S., Lin B., Louder M.K., McKee K., O’Dell S. (2018). Surface-Matrix Screening Identifies Semi-specific Interactions that Improve Potency of a Near Pan-reactive HIV-1-Neutralizing Antibody. Cell Rep..

[122] Rujas E., Leaman D.P., Insausti S., Ortigosa-Pascual L., Zhang L., Zwick M.B., Nieva J.L. (2018). Functional Optimization of Broadly Neutralizing Hiv-1 Antibody 10e8 by Promoting Membrane Interactions. J. Virol..

[123] Rujas E., Insausti S., Leaman D.P., Carravilla P., Gonzalez-Resines S., Monceaux V., Sanchez-Eugenia R., Garcia-Porras M., Iloro I., Zhang L. (2020). Affinity for the Interface Underpins Potency of Antibodies Operating In Membrane Environments. Cell Rep..

[124] Yang G., Holl T.M., Liu Y., Li Y., Lu X., Nicely N.I., Kepler T.B., Alam S.M., Liao H.X., Cain D.W. (2013). Identification of autoantigens recognized by the 2F5 and 4E10 broadly neutralizing HIV-1 antibodies. J. Exp. Med..

[125] Liu M., Yang G., Wiehe K., Nicely N.I., Vandergrift N.A., Rountree W., Bonsignori M., Alam S.M., Gao J., Haynes B.F. (2015). Polyreactivity and autoreactivity among HIV-1 antibodies. J. Virol..

[126] Verkoczy L., Kelsoe G., Haynes B.F. (2014). HIV-1 envelope gp41 broadly neutralizing antibodies: Hurdles for vaccine development. PLoS Pathog..

[127] Nemazee D. (2017). Mechanisms of central tolerance for B cells. Nat. Rev. Immunol..

[128] Verkoczy L., Chen Y., Zhang J., Bouton-Verville H., Newman A., Lockwood B., Scearce R.M., Montefiori D.C., Dennison S.M., Xia S.M. (2013). Induction of HIV-1 broad neutralizing antibodies in 2F5 knock-in mice: Selection against membrane proximal external region-associated autoreactivity limits T-dependent responses. J. Immunol..

[129] Doyle-Cooper C., Hudson K.E., Cooper A.B., Ota T., Skog P., Dawson P.E., Zwick M.B., Schief W.R., Burton D.R., Nemazee D. (2013). Immune tolerance negatively regulates B cells in knock-in mice expressing broadly neutralizing HIV antibody 4E10. J. Immunol..

[130] Verkoczy L., Chen Y., Bouton-Verville H., Zhang J., Diaz M., Hutchinson J., Ouyang Y.B., Alam S.M., Holl T.M., Hwang K.K. (2011). Rescue of HIV-1 broad neutralizing antibody-expressing B cells in 2F5 VH x VL knockin mice reveals multiple tolerance controls. J. Immunol..

[131] Zhang R., Verkoczy L., Wiehe K., Munir Alam S., Nicely N.I., Santra S., Bradley T., Pemble C.W.t., Zhang J., Gao F. (2016). Initiation of immune tolerance-controlled HIV gp41 neutralizing B cell lineages. Sci. Transl. Med..

[132] Zhu J., Ofek G., Yang Y., Zhang B., Louder M.K., Lu G., McKee K., Pancera M., Skinner J., Zhang Z. (2013). Mining the antibodyome for HIV-1-neutralizing antibodies with next-generation sequencing and phylogenetic pairing of heavy/light chains. Proc. Natl. Acad. Sci. USA.

[133] Soto C., Ofek G., Joyce M.G., Zhang B., McKee K., Longo N.S., Yang Y., Huang J., Parks R., Eudailey J. (2016). Developmental Pathway of the MPER-Directed HIV-1-Neutralizing Antibody 10E8. PLoS ONE.

[134] Arnaout R., Lee W., Cahill P., Honan T., Sparrow T., Weiand M., Nusbaum C., Rajewsky K., Koralov S.B. (2011). High-resolution description of antibody heavy-chain repertoires in humans. PLoS ONE.

[135] Klein F., Diskin R., Scheid J.F., Gaebler C., Mouquet H., Georgiev I.S., Pancera M., Zhou T., Incesu R.B., Fu B.Z. (2013). Somatic mutations of the immunoglobulin framework are generally required for broad and potent HIV-1 neutralization. Cell.

[136] Torralba J., de la Arada I., Carravilla P., Insausti S., Rujas E., Largo E., Eggeling C., Arrondo J.L.R., Apellaniz B., Nieva J.L. (2020). Cholesterol Constrains the Antigenic Configuration of the Membrane-Proximal Neutralizing HIV-1 Epitope. ACS Infect. Dis..

[137] Shao S., Huang W.C., Lin C., Hicar M.D., LaBranche C.C., Montefiori D.C., Lovell J.F. (2020). An Engineered Biomimetic MPER Peptide Vaccine Induces Weakly HIV Neutralizing Antibodies in Mice. Ann. Biomed. Eng..

[138] Zhang Z., Wei X., Lin Y., Huang F., Shao J., Qi J., Deng T., Li Z., Gao S., Li S. (2019). HIV-1 Membrane-Proximal External Region Fused to Diphtheria Toxin Domain-A Elicits 4E10-Like Antibodies in Mice. Immunol. Lett..

[139] Sun Z., Zhu Y., Wang Q., Ye L., Dai Y., Su S., Yu F., Ying T., Yang C., Jiang S. (2016). An immunogen containing four tandem 10E8 epitope repeats with exposed key residues induces antibodies that neutralize HIV-1 and activates an ADCC reporter gene. Emerg. Microbes Infect..

[140] Banerjee S., Shi H., Habte H.H., Qin Y., Cho M.W. (2016). Modulating immunogenic properties of HIV-1 gp41 membrane-proximal external region by destabilizing six-helix bundle structure. Virology.

[141] Donius L.R., Cheng Y., Choi J., Sun Z.Y., Hanson M., Zhang M., Gierahn T.M., Marquez S., Uduman M., Kleinstein S.H. (2016). Generation of Long-Lived Bone Marrow Plasma Cells Secreting Antibodies Specific for the HIV-1 gp41 Membrane-Proximal External Region in the Absence of Polyreactivity. J. Virol..

[142] Yi G., Tu X., Bharaj P., Guo H., Zhang J., Shankar P., Manjunath N. (2015). Human Rhinovirus Presenting 4E10 Epitope of HIV-1 MPER Elicits Neutralizing Antibodies in Human ICAM-1 Transgenic Mice. Mol. Ther..

[143] Krebs S.J., McBurney S.P., Kovarik D.N., Waddell C.D., Jaworski J.P., Sutton W.F., Gomes M.M., Trovato M., Waagmeester G., Barnett S.J. (2014). Multimeric scaffolds displaying the HIV-1 envelope MPER induce MPER-specific antibodies and cross-neutralizing antibodies when co-immunized with gp160 DNA. PLoS ONE.

[144] Strasz N., Morozov V.A., Kreutzberger J., Keller M., Eschricht M., Denner J. (2014). Immunization with hybrid proteins containing the membrane proximal external region of HIV-1. AIDS Res. Hum. Retrovir..

[145] Mohan T., Verma P., Rao D.N. (2014). Comparative mucosal immunogenicity of HIV gp41 membrane-proximal external region (MPER) containing single and multiple repeats of ELDKWA sequence with defensin peptides. Immunobiology.

[146] Zhai Y., Zhong Z., Zariffard M., Spear G.T., Qiao L. (2013). Bovine papillomavirus-like particles presenting conserved epitopes from membrane-proximal external region of HIV-1 gp41 induced mucosal and systemic antibodies. Vaccine.

[147] Dawood R., Benjelloun F., Pin J.J., Kone A., Chanut B., Jospin F., Lucht F., Verrier B., Moog C., Genin C. (2013). Generation of HIV-1 potent and broad neutralizing antibodies by immunization with postfusion HR1/HR2 complex. AIDS.

[148] Bomsel M., Tudor D., Drillet A.S., Alfsen A., Ganor Y., Roger M.G., Mouz N., Amacker M., Chalifour A., Diomede L. (2011). Immunization with HIV-1 gp41 subunit virosomes induces mucosal antibodies protecting nonhuman primates against vaginal SHIV challenges. Immunity.

[149] Dennison S.M., Sutherland L.L., Jaeger F.H., Anasti K.M., Parks R., Stewart S., Bowman C., Xia S.M., Zhang R., Shen X. (2011). Induction of Antibodies in Rhesus Macaques That Recognize a Fusion-Intermediate Conformation of HIV-1 gp41. PLoS ONE.

[150] Hinz A., Schoehn G., Quendler H., Hulsik D.L., Stiegler G., Katinger H., Seaman M.S., Montefiori D., Weissenhorn W. (2009). Characterization of a trimeric MPER containing HIV-1 gp41 antigen. Virology.

[151] Mantis N.J., Kozlowski P.A., Mielcarz D.W., Weissenhorn W., Neutra M.R. (2001). Immunization of mice with recombinant gp41 in a systemic prime/mucosal boost protocol induces HIV-1-specific serum IgG and secretory IgA antibodies. Vaccine.

